# B Cell Receptor-Responsive miR-141 Enhances Epstein-Barr Virus Lytic Cycle via FOXO3 Inhibition

**DOI:** 10.1128/mSphere.00093-21

**Published:** 2021-04-14

**Authors:** Yan Chen, Devin N. Fachko, Nikita S. Ivanov, Rebecca L. Skalsky

**Affiliations:** a Vaccine and Gene Therapy Institute, Oregon Health & Science University, Beaverton, Oregon, USA; University of Arizona

**Keywords:** Epstein-Barr virus, FOXO3, herpesviruses, latency, lymphoma, microRNA

## Abstract

Antigen recognition by the B cell receptor (BCR) is a physiological trigger for reactivation of Epstein-Barr virus (EBV) and can be recapitulated *in vitro* by cross-linking of surface immunoglobulins. Previously, we identified a subset of EBV microRNAs (miRNAs) that attenuate BCR signal transduction and subsequently dampen lytic reactivation in B cells. The roles of host miRNAs in the EBV lytic cycle are not completely understood. Here, we profiled the small RNAs in reactivated Burkitt lymphoma cells and identified several miRNAs, such as miR-141, that are induced upon BCR cross-linking. Notably, EBV encodes a viral miRNA, miR-BART9, with sequence homology to miR-141. To better understand the functions of these two miRNAs, we examined their molecular targets and experimentally validated multiple candidates commonly regulated by both miRNAs. Targets included B cell transcription factors and known regulators of EBV immediate-early genes, leading us to hypothesize that these miRNAs modulate kinetics of the lytic cascade in B cells. Through functional assays, we identified roles for miR-141 and EBV miR-BART9 and one specific target, FOXO3, in progression of the lytic cycle. Our data support a model whereby EBV exploits BCR-responsive miR-141 and further mimics activity of this miRNA family via a viral miRNA to promote productive lytic replication.

**IMPORTANCE** EBV is a human pathogen associated with several malignancies. A key aspect of lifelong virus persistence is the ability to switch between latent and lytic replication modes. The mechanisms governing latency, reactivation, and progression of the lytic cycle are only partly understood. This study reveals that specific miRNAs can act to support the EBV lytic phase following BCR-mediated reactivation triggers. Furthermore, this study identifies a role for FOXO3, commonly suppressed by both host and viral miRNAs, in modulating progression of the EBV lytic cycle.

## INTRODUCTION

Epstein-Barr virus (EBV) is a ubiquitous gammaherpesvirus closely associated with lymphoproliferative diseases in immunocompromised individuals, and linked to cancers such as Hodgkin and non-Hodgkin lymphomas, nasopharyngeal carcinoma, and Burkitt lymphoma (BL) (1, 2). BL is a common childhood cancer in Africa with >80% of cases positive for EBV (3). As a germinal-center (GC)-derived cancer, BL cells exhibit a centroblast-like phenotype (2, 4), maintained in part from activated c-*myc* oncogene expression (5), and have a molecular profile akin to GC dark zone proliferative B cells (6).

EBV has both a latent and a lytic replication phase, and latent EBV periodically reactivates to produce infectious virions. The EBV lytic stage is essentially required for horizontal transmission and lifelong persistence and has a poorly understood role in the development of viral malignancies (7). In addition to epithelial cells in the oropharynx (8), resting peripheral blood and tonsil memory B cells are thought to serve as reservoirs for latent EBV (2, 9), while mature B cell trafficking through the GC and terminal differentiation into CD38^+^ plasma cells can trigger EBV reactivation (10). *In vitro*, cross-linking of surface immunoglobulins (Igs) on freshly isolated EBV-positive B cells (11) or latently infected BL cells (12) functionally mimics antigen interactions and stimulates virus reactivation. The complex signaling events initiated following cross-linking of the B cell receptor (BCR) induces expression of EBV immediate-early (IE) genes BZLF1 and BRLF1, which subsequently transactivate early and late genes, such as BMRF1/EA-D, and other viral products involved in the synthesis of viral genomes, virion assembly, and egress during the lytic cycle (7).

Exact molecular mechanisms, such as underlying posttranscriptional processes, that control EBV latency, reactivation, and progression of the lytic cycle remain to be fully elucidated (7, 13). Master transcriptional regulators of plasma cell differentiation, including Blimp-1/PRDM1, can activate the EBV Zta/BZLF1 (Zp) and Rta/BRLF1 (Rp) promoters (13, 14). Transcription factors such as ATF, Sp1/3, MEF2D, XBPs, CREB family members, AP1 heterodimers (i.e., c-Jun), and HIF1a interact with Zp in response to antigen stimulation or oxidative stress; Zp further contains *cis*-regulatory elements which confer autoregulation (7, 13, 15–18). Repressors of Zp include the zinc-finger E-box-binding proteins encoded by ZEB1 and ZEB2 and the polycomb protein Yin Yang 1 (YY1) (7, 13, 19–21). Notably, microRNAs (miRNAs) from the miR-200 family (miR-200b and miR-429 expressed in epithelial cells) posttranscriptionally silence ZEB1/2 expression, leading to enhanced EBV reactivation through increased Zp activity (22, 23).

miRNAs are ∼22 nucleotide (nt) noncoding RNAs that posttranscriptionally control gene expression and regulate multiple biological processes, including B cell development, GC reactions, and the progression of immune responses (24, 25). Deregulated miRNA activity is implicated in B cell lymphomagenesis, and normal B cell subtypes, as well as B cell cancers, can be distinguished by miRNA signatures (24, 26). EBV encodes >44 viral miRNAs, the majority of which are expressed from the BART locus, and exhibit expression kinetics similar to the BART transcripts (27). BART miRNAs are detectable throughout phases of EBV infection, including latency I (28), suggesting these molecules actively facilitate maintenance of the latent state (29, 30). In addition, viral miRNAs are rapidly induced upon entry into the lytic cycle (27, 28), indicating further roles in EBV infection processes. Recently, we identified several EBV miRNAs that attenuate BCR signal transduction and consequently dampen BCR-induced lytic gene expression, demonstrating that a subset of the viral miRNAs can actively counter EBV reactivation triggers (31).

The functions for host miRNAs in EBV reactivation and progression of the lytic cycle are not well characterized. Subversion of apoptosis and evasion of antiviral responses are key parts of productive virus replication, and several groups have demonstrated roles for miRNAs in these processes (30, 32). In addition, studies in which Dicer was inhibited reported reduced levels of IE gene expression following EBV reactivation, providing evidence that components of the miRNA biogenesis machinery are necessary for aspects of the lytic phase (33, 34). We previously reported that disruption of cellular miR-17 in EBV-positive BL cells augments IE gene expression (31), and other groups have shown that miR-200 family members expressed in epithelial cells have essential roles in the EBV latent to lytic switch (22, 23). Here, we investigated how EBV exploits BCR-responsive miRNAs to navigate the lytic replication phase. Specifically, we aimed (i) to define the miRNAs that are impacted by BCR cross-linking, (ii) to elucidate targets of those miRNAs, and (iii) to determine whether the BCR-responsive miRNAs and/or their targets have functional roles in initial reactivation events and/or progression of the EBV lytic cycle.

## RESULTS

### BCR-mediated EBV reactivation induces changes in cellular miRNA levels.

**FIG 1:**
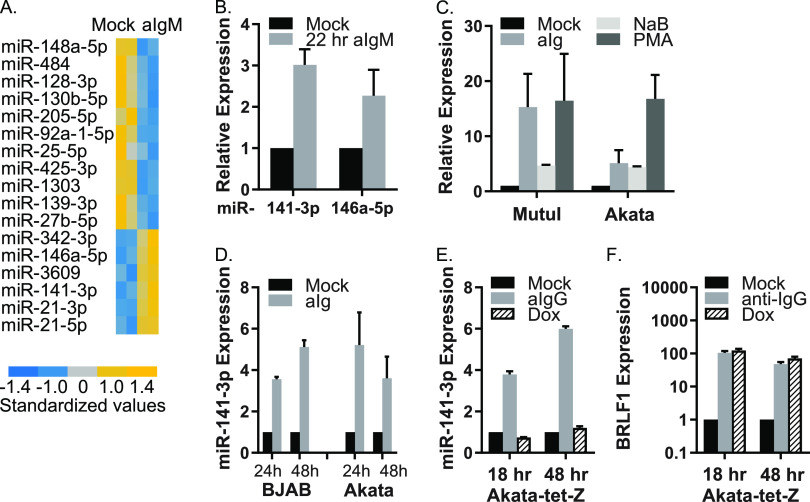
miRNA signatures in response to BCR cross-linking. (A) Heatmap of significant DE cellular miRNAs in anti-IgM-treated MutuI cells compared to mock-treated cells (*P* < 0.05, false discovery rate [FDR] < 0.05, read count [RC] > 20). Values for each miRNA are standardized across samples to a mean = 0 and SD ± 1. (B) TaqMan qRT-PCR analysis of cellular miRNA expression in MutuI cells treated with 2.5 μg/ml anti-IgM for 22 h. (C) TaqMan qRT-PCR analysis of miR-141-3p in MutuI or Akata cells treated with the indicated agents for 48 h. The averages from two independent experiments are shown. (D and E) TaqMan qRT-PCR analysis of miR-141-3p in anti-IgM-treated EBV-negative BJAB cells, anti-IgG-treated EBV-positive Akata cells, or anti-IgG or doxycycline (Dox)-treated Akata-tet-Z cells. (F) Expression of EBV IE gene BRLF1 in Akata-tet-Z cells after anti-IgG or Dox treatment. BRLF1 levels were assayed by qRT-PCR at the times indicated. Values are normalized to GAPDH and shown relative to mock-treated cells. Unless otherwise stated, in Fig. 1, miRNA expression values are normalized to cellular miR-16 and reported relative to levels in mock-treated cells. Averages and SD are shown from at least three independent experiments.

To investigate the miRNA response to BCR cross-linking, we treated EBV-positive MutuI cells with antibodies to surface Ig (aIgM) for 22 h and profiled the small RNAs by deep sequencing (<200 nucleotides [nt]). Over one million sequences were obtained per sample and, after alignment to the human and EBV genomes, 1,015 distinct miRNAs (read count >1 in at least one library) were identified. Of these, 392 mature miRNAs were considered expressed (read counts ≥10) and used to determine differential expression (DE) in mock-treated versus anti-IgM-treated cells (Fig. 1A). Consistent with prior reports (27, 28), nearly all EBV miRNAs were upregulated after anti-IgM treatment (see Fig. S1A and B in the supplemental material). EBV miRNAs not shown (i.e., miR-BHRF1-2-5p) were still detected, but at levels below our cutoffs. Significant changes were observed for several host miRNAs. Notably, miR-141-3p, miR-146a-5p, miR-342-3p, miR-3609, miR-21-3p, and miR-21-5p increased upon BCR stimulation, while 11 miRNAs, including miR-148a-5p, miR-27b-5p, and miR-139-3p, significantly decreased (Fig. 1A). BCR-mediated induction of miR-141-3p and miR-146a-5p were independently verified by quantitative reverse transcription-PCR (qRT-PCR) (Fig. 1B; see also Fig. S1B). We measured levels of other miRNAs which have been linked to BCR stimulation and/or EBV reactivation (i.e., miR-17/92, miR-181, and miR-190) (35, 36) but did not detect robust changes in their expression (see Fig. S1C).

**FIG 2:**
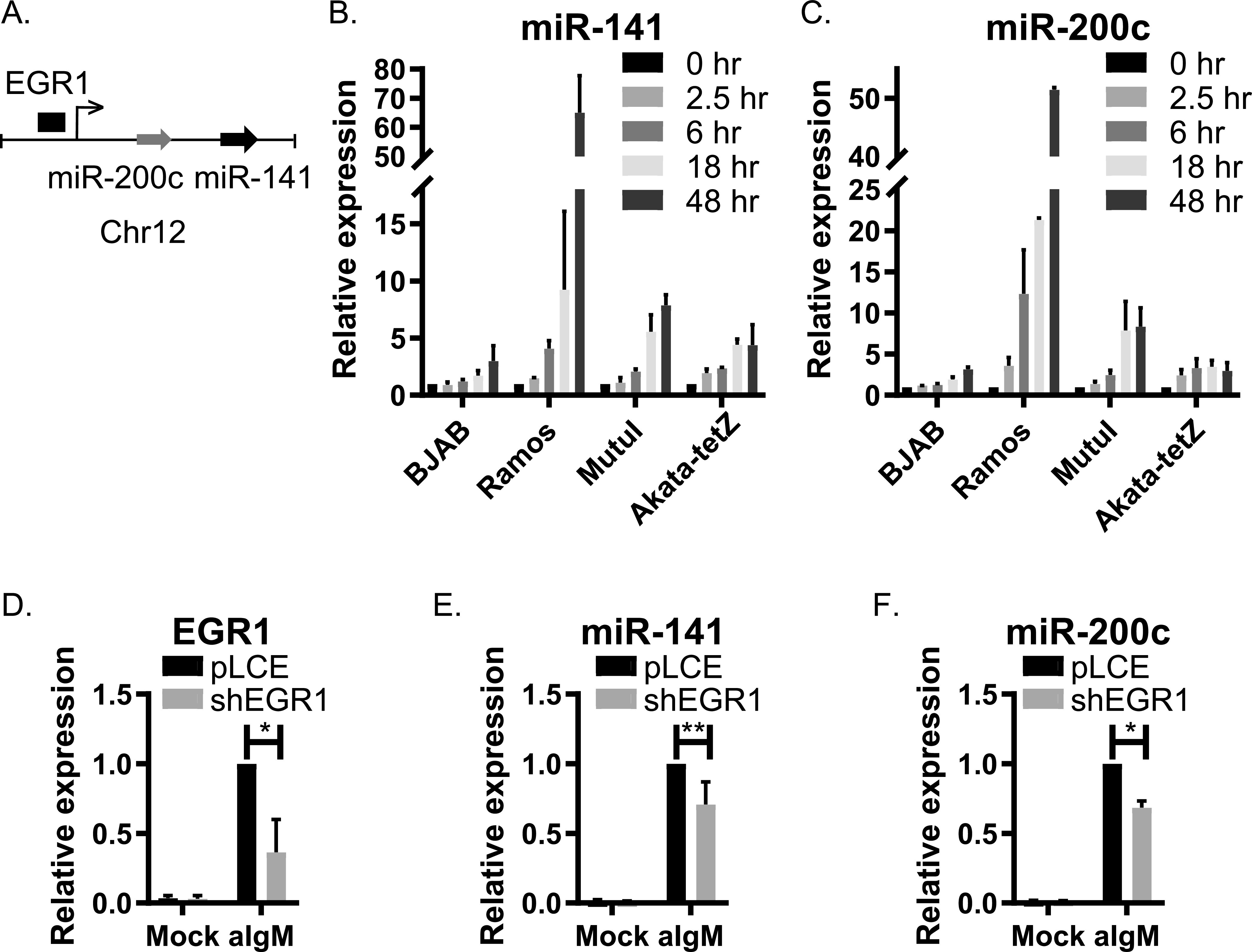
miR-141 and miR-200c accumulate in response to BCR signaling. (A) Schematic illustration of human chromosome 12 harboring the miR-200c/141 locus. (B and C) TaqMan qRT-PCR analysis of expression of miR-141 and miR-200c at the indicated time points after anti-Ig treatment of EBV-negative cells (BJAB and Ramos) and EBV-positive cells (MutuI and Akata-tet-Z). Values are normalized to miR-16 and reported relative to levels at 0 h in each respective cell line. (D) shRNA knockdown of EGR1 in Ramos cells assayed by qRT-PCR analysis. Expression levels are normalized to GAPDH and reported relative to anti-IgM-treated control (pLCE) cells. (E and F) Induction of miR-141 and miR-200c in Ramos cells by BCR cross-linking is impaired by EGR1 knockdown. miRNA expression levels were analyzed by TaqMan qRT-PCR. Values are normalized to miR-16 and reported relative to levels in anti-IgM-treated control (pLCE) cells. Averages and SD are shown from at least three independent experiments. **, *P* < 0.05; *, *P* < 0.01 (Student *t* test).

10.1128/mSphere.00093-21.1 FIG S1 miRNA expression levels in response to surface Ig cross-linking. (A) Heatmap of significant DE EBV miRNAs in anti-IgM-treated MutuI cells compared to mock-treated cells (*P* < 0.05, FDR < 0.05, RC > 20). Values for each miRNA are standardized across samples to a mean = 0 and SD ± 1. (B and C) TaqMan qRT-PCR analysis of indicated cellular and EBV miRNAs in anti-IgM-treated MutuI cells. Values are normalized to miR-16 and reported relative to levels in mock-treated cells. (D) Schematic illustration of human chromosome 1 harboring the miR-200b/a/429 locus. (E and F) TaqMan qRT-PCR analysis of expression of miR-429 and miR-200b at 48 h after anti-Ig treatment of the same panel of B cells as in Fig. 2. Values are normalized to miR-16 and reported relative to levels in each respective mock-treated cell line. Shown are the averages and SD from at least three independent experiments. Download FIG S1, EPS file, 1.6 MB. Copyright © 2021 Chen et al. 2021 Chen et al. https://creativecommons.org/licenses/by/4.0/ This content is distributed under the terms of the Creative Commons Attribution 4.0 International license.

### miR-141/200c is responsive to BCR cross-linking irrespective of EBV infection status.

Enhanced miR-146a levels have been linked to LMP1 expression (37), while miR-21 is upregulated after *de novo* EBV infection (38) and in the presence of EBNA2 (39). miR-342-3p is BCR-responsive in murine WEHI-231 cells (40). We therefore selected miR-141-3p for further analysis. While miR-141-3p has been studied extensively in epithelial cells, less is known about this miRNA in B cells. We assayed miRNA expression in both MutuI and Akata cells treated with various reactivation agents, including sodium butyrate (NaB), a histone deacetylase inhibitor, and phorbol 12-myristate 13-acetate (PMA), an activator of protein kinase C. In addition to BCR cross-linking, miR-141-3p was upregulated in response to both NaB and PMA (Fig. 1C).

To investigate whether miR-141-3p induction is linked to any EBV factors, we examined additional BL cell lines. Surface Ig cross-linking increased miR-141-3p in both EBV-negative and EBV-positive cells (Fig. 1D and Fig. 2B), suggesting that miR-141-3p induction is not dependent upon EBV but is instead a cellular response to BCR signals. To definitively rule out EBV as a contributor, we tested miR-141-3p levels in EBV^+^ BL cells expressing a doxycycline-inducible Zta (Akata-tet-Z) (41). In these cells, EBV reactivation can be initiated through the BCR or, alternatively, through direct chemical induction of Zta. Treatment of Akata-tet-Z cells with anti-IgG led to increased miR-141-3p similar to what was observed for other BL cells; however, when EBV lytic replication was activated independently of the BCR, miR-141-3p levels were unaffected (Fig. 1E and F). We therefore conclude that miR-141-3p induction is linked to the cell signaling response initiated through BCR engagement and not mediated through any viral gene products.miR-141 is part of the miR-200 family, consisting of five members that are processed from polycistronic primary miRNA transcripts (Fig. 2A; see also Fig. S1D) (42). While levels of other miR-200 family members were below our stringent cutoffs for miRNA profiling, we did observe modest increases in miR-200c in response to anti-IgM (not shown), suggesting that the miR-141/200c pri-miRNA is transcriptionally activated and processed. To explore this further, miR-200c, miR-429, and miR-200b levels were monitored in BL cells. Both miR-141-3p and miR-200c temporally accumulated with similar kinetics (Fig. 2B and C), consistent with activation of the pri-miRNA. In contrast, miR-429 and miR-200b were induced only in MutuI cells, indicating alternate modes of regulation (see Fig. S1E and F).

The miR-141/200c promoter contains response elements for EGR1 (43), a rapid response nuclear zinc finger transcription factor that is upregulated upon BCR engagement (44). To examine EGR1 as a possible mechanism for how miR-141 and miR-200c are induced, we inhibited EGR1 with short hairpin RNAs (shRNAs) (Fig. 2D). Knockdown of EGR1 attenuated but did not fully abrogate miR-141 and miR-200c induction upon BCR cross-linking (Fig. 2E and F), pointing to a partial role for EGR1 in miR-141/200c expression.

### miR-141 activity contributes to the EBV lytic phase.

**FIG 3:**
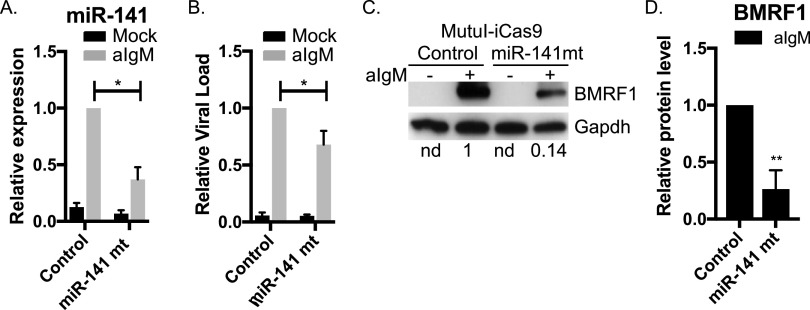
miR-141 contributes to efficient EBV lytic reactivation. (A) TaqMan qRT-PCR analysis of miR-141 in MutuI-iCas9 confirms significant knockdown of miR-141. Values are normalized to miR-16 and reported relative to levels of anti-IgM-treated (48 h) empty gRNA control cells. *, *P* < 0.05 (Student *t* test). (B) Genomic DNA was isolated from mock-treated or anti-IgM-treated MutuI-iCas9 cells stably transduced with either empty gRNA (control) or gRNA against miR-141 (miR-141 mt). Viral loads were determined by qPCR assay using primers to the LMP1 region. Values are normalized to GAPDH and reported relative to viral levels in anti-IgM-treated empty gRNA control cells. Averages from four independent experiments are shown. *, *P* < 0.05 (Student *t* test). (C) Immunoblot of BMRF1 lytic gene product, Ea-D, in mock-treated or anti-IgM-treated MutuI-iCas9 cells stably transduced with either empty gRNA or gRNA against miR-141. GAPDH levels are shown as loading controls. Band intensities were quantified using ImageJ, normalized to loading controls, and reported relative to anti-IgM-treated empty gRNA control cells. The results of one of four representative experiments are shown. (D) Quantification of immunoblot of MutuI-iCas9 for BMRF1 gene product (*n* = 4). **, *P* < 0.01 (Student *t* test).

Prior studies in epithelial cells demonstrated positive correlations between EBV lytic gene expression and miR-200 family members (22, 23). To determine whether miR-141 plays a role in the EBV reactivation process in B cells, we generated MutuI-iCas9 cells expressing a tet-inducible Cas9 and introduced guide RNAs (gRNAs) to genetically inactivate miR-141. After doxycycline treatment to activate Cas9, miRNA levels were measured by qRT-PCR, confirming specific disruption of miR-141 but not disruption of miR-200c (Fig. 3A; see also Fig. S2A). We subsequently tested EBV reactivation. Compared to control cells transduced with empty vector, CRISPR-mediated disruption of miR-141 yielded significant decreases in viral loads following surface Ig cross-linking (Fig. 3B). We measured levels of the EBV BMRF1/Ea-D protein, a nuclear antigen essential for viral genome synthesis during lytic replication. Consistent with reduced viral loads, we observed attenuated BMRF1 expression upon perturbation of miR-141 function (Fig. 3C and D). These results clearly demonstrate that miR-141 activity conferred through BCR-mediated signaling contributes to the EBV lytic infection cycle.

10.1128/mSphere.00093-21.2 FIG S2 TaqMan qRT-PCR analysis of miR-200c in MutuI-iCas9 (A), Ramos-iCas9 (B), and Akata-iCas9 (C) cells confirms that miR-200c expression is not impaired by miR-141 knockdown (see Fig. 3 and 5). Values are normalized to miR-16 and reported relative to levels of anti-IgM-treated empty gRNA control cells. Download FIG S2, EPS file, 1.1 MB. Copyright © 2021 Chen et al. 2021 Chen et al. https://creativecommons.org/licenses/by/4.0/ This content is distributed under the terms of the Creative Commons Attribution 4.0 International license.

### miR-141-3p and miR-BART9-3p regulate common cellular targets.

**FIG 4:**
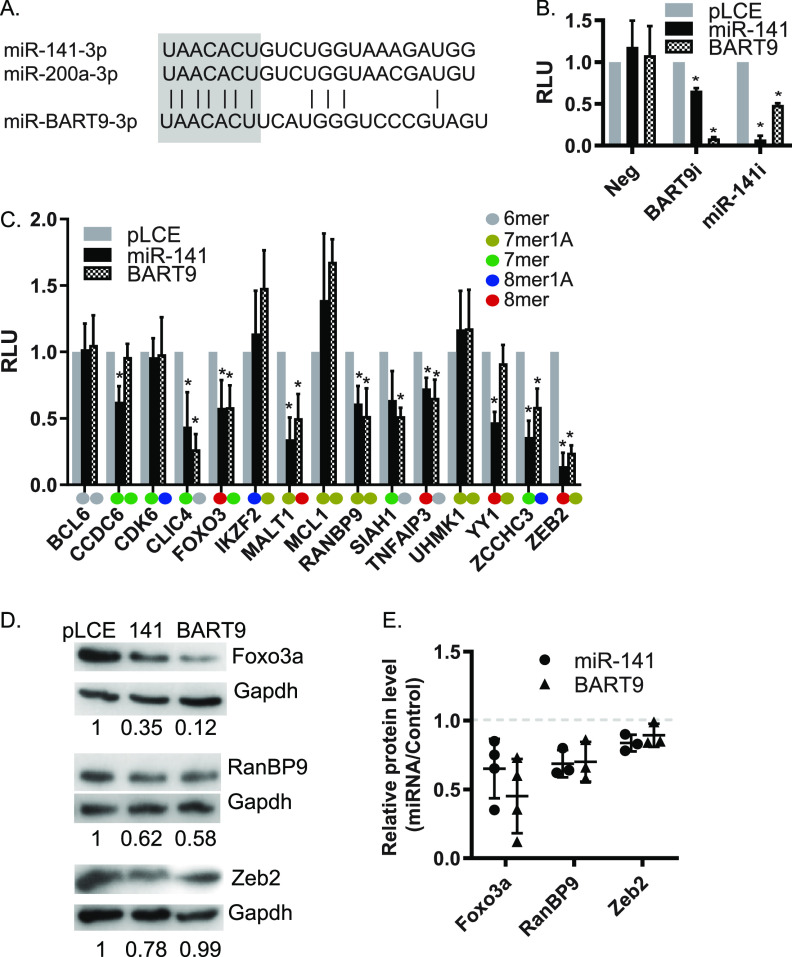
miR-141-3p and EBV miR-BART9-3p regulate 3′ UTRs through common seed interactions. (A) Alignment of miR-141-3p and miR-200a-3p with ebv-miR-BART9-3p. The sequence homology within the seed regions (nt 1 to 7) of these miRNAs is highlighted. (B) miRNA expression vectors (pLCE or BART9 or miR-141) were cotransfected into HEK293T cells with reporters harboring perfect binding sites in the luciferase 3′ UTR for either miR-BART9-3p (BART9i) or miR-141-3p (miR-141i). At 48 h posttransfection, cells were lysed in 1× passive lysis buffer. The luciferase activity was measured using a dual luciferase reporter kit. Values are reported relative to empty reporter in control (pLCE) cells. Shown are the averages from three independent experiments performed in triplicate. *, *P* < 0.05 (Student *t* test). RLU, relative light units. (C) Luciferase reporter assays confirm common 3′ UTRs targeted by miR-141 and miR-BART9. HEK293T cells were cotransfected with psiCheck2 vector harboring selected 3′ UTR luciferase reporters and EBV miRNA expression vectors (pLCE-based). At 48 to 72 h posttransfection, cells were lysed and assayed for dual luciferase activity. Shown are the averages from at least three experiments performed in triplicate. *, *P* < 0.05 (Student *t* test). RLU, relative light units. The type of seed match between each individual miRNA and 3′ UTR are labeled in different colors. (D and E) Representative immunoblots and quantification of immunoblots for Foxo3a, RanBP9, and Zeb2 in HEK293T cells transfected with miRNA expression vectors. Gapdh is shown as a loading control. Band intensities were quantified using ImageJ, normalized to loading controls, and reported relative to empty vector (pLCE) control cells. Reported are the results of three (RanBP9 and Zeb2) or four (Foxo3a) independent experiments.

Intriguingly, EBV encodes a viral miRNA, miR-BART9-3p, which exhibits seed sequence homology to miR-141-3p (Fig. 4A), leading to the hypothesis that the viral and cellular miRNAs have common targets and common activities mediated through interactions with cognate seed match sites. To formally test this idea, miR-141 and miR-BART9 expression vectors were cotransfected with reporters harboring perfect binding sites in the luciferase 3′ untranslated region (3′ UTR) for either miR-BART9-3p or miR-141-3p (Fig. 4B). As expected, both the viral and cellular miRNA potently downregulated their associated reporters. Notably, we observed ∼40% knockdown of luciferase activity when miR-141 was tested against the miR-BART9 reporter and ∼60% knockdown when miR-BART9 was tested against the miR-141 reporter (Fig. 4B). Since the only stretch of sequence homology between these two miRNAs is the 5′ seed region (nt 1 to 7), these results demonstrate that miR-141-3p and miR-BART9-3p are capable of functionally interacting with target RNAs through common 5′ sequences.

To investigate biological targets of miR-141-3p and miR-BART9-3p, we next assembled a list of native 3′ UTR interactions extracted from previously published studies (45–49). Target interactions were assigned based upon canonical miRNA seed pairing (i.e., nt 2 to 7). Additional candidates predicted by TargetScan (50) were included for a comprehensive list (see Fig. S3A). We identified 1,880 unique 3′ UTR targets harboring canonical 5′ seed matches to miR-141-3p, miR-200a, and/or miR-BART9-3p, of which 321 targets overlapped with one or more study. Comparison of the 187 3′ UTRs captured in PAR-CLIP studies revealed that 41% of interaction sites could be assigned to both miR-BART9-3p and miR-141-3p based upon the presence of seed-match sites (Fig. S3A, inset).miRNA interaction sites in CLIP and RNAseq studies are assigned computationally; thus, to experimentally confirm targets, luciferase reporters were constructed for 14 3′ UTRs. We specifically selected 3′ UTRs of interest to either B cell or EBV biology. These included (i) FOXO3, encoding Forkhead box O3a, a member of the conserved forkhead box transcription factors (51); (ii) RANBP9, encoding an adaptor protein that interacts with Zta to augment reactivation (52); (iii) YY1, necessary for GC reactions (53) and binds multiple sites within Zp to repress Zta expression (21); (iv) ZCCHC3, encoding a cosensor for cGAS (54); and (v) ZEB2, a transcriptional repressor of Zp (13, 22). Binding sites in these 3′ UTRs represented a range of 6mer (nt 2 to 7) to 8mer (nt 2 to 9) seed matches for miR-141-3p and/or miR-BART9-3p (Fig. 4C).

10.1128/mSphere.00093-21.3 FIG S3 (A) Overlap of miRNA interactions identified from published Ago-CLIP and RNA-Seq datasets and predicted by TargetScan. Reported are 3′ UTR interactions with >7mer seed match to either miR-BART9-3p or miR-141-3p. (B) qRT-PCR analysis of miRNA targets. 293-EBV2089 cells were transfected with pLCE, pLCE-miR-141, or pLCE-BART9 as indicated. At 48 h posttransfection, RNA was harvested and analyzed for gene expression. Values are normalized to GAPDH and reported relative to the levels of pLCE control cells. Shown are the averages from three independent experiments. *, *P* < 0.05 (Student *t* test). Download FIG S3, EPS file, 2.1 MB. Copyright © 2021 Chen et al. 2021 Chen et al. https://creativecommons.org/licenses/by/4.0/ This content is distributed under the terms of the Creative Commons Attribution 4.0 International license.

Reporters were tested in HEK293T cells cotransfected with miRNA expression vectors. For seven 3′ UTRs, we observed significant inhibition of luciferase activity in the presence of either miRNA, supporting the idea that miR-BART9-3p functional mimics miR-141-3p (Fig. 4C). Interestingly, luciferase assays further revealed distinct targeting of CCDC6 and YY1 3′ UTRs by miR-141 but not miR-BART9 (Fig. 4C). Presumably, these targeting differences are due to 3′ compensatory binding.

We subsequently assessed the impact of miR-141 and miR-BART9 on endogenous targets. Ectopic expression of miR-141 in EBV-infected 293 cells significantly reduced the steady-state RNA levels of RANBP9 and ZEB2 (see Fig. S3B). Moreover, we observed reduced Foxo3a, RanBP9, and Zeb2 protein levels in HEK293T cells in the presence of miR-141 (Fig. 4D and E). Notably, both Foxo3a and RanBP9 protein levels were also significantly reduced in response to ectopic miR-BART9 (Fig. 4D and E). Surprisingly, despite the ZEB2 3′ UTR and ZEB2 RNA levels responding to both miRNAs (Fig. 4C; see also Fig. S3B), we detected decreases in Zeb2 protein only in the presence of miR-141 (Fig. 4D), suggesting that ZEB2 may not be targeted by miR-BART9. Taken together, these experiments formally demonstrate that EBV miR-BART9-3p partly mimics miR-141-3p activity and regulates common cellular targets such as RANBP9 and FOXO3.

### miR-141 regulates Foxo3a expression in BL cells.

**FIG 5:**
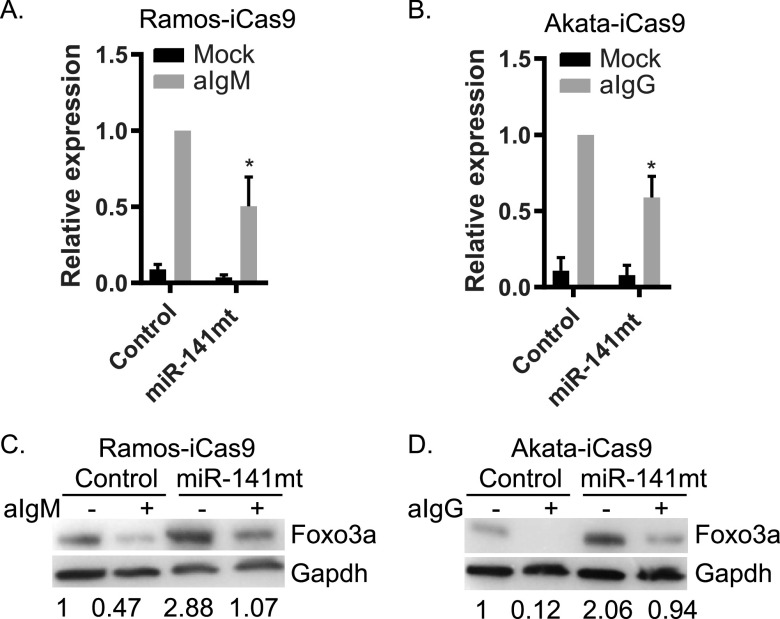
miR-141 regulates Foxo3a levels in BL cells. (A and B) TaqMan qRT-PCR analysis of miR-141 in Ramos-iCas9 and Akata-iCas9 confirms knockdown of miR-141. Values are normalized to miR-16 and reported relative to levels of anti-IgM-treated empty gRNA control cells. *, *P* < 0.05 (Student *t* test). (C and D) Immunoblot for Foxo3a in Ramos-iCas9 and Akata-iCas9 cells stably transduced with either empty gRNA (Control) or gRNA against miR-141 (miR-141 mt). Gapdh levels are shown as loading controls. Band intensities were quantified using ImageJ, normalized to loading controls, and reported relative to mock-treated empty gRNA control cells. Shown are representative results for one of two independent experiments.

FOXO transcription factors are important stress-responsive components linked to lymphocyte homeostasis and cancer immunity (51, 55). Previous studies demonstrate that FOXO3 is posttranscriptionally regulated by multiple miRNAs, including miR-155, miR-132, miR-212, miR-27a, and miR-96 (56). Having observed that Foxo3a levels are responsive to ectopic miR-141 in HEK293T cells, we sought to verify that FOXO3 is indeed directly targeted by miR-141 in BL cells. We used a lentiviral CRISPR-based system to disrupt endogenous miR-141 expression in EBV-negative Ramos-iCas9 and EBV-positive Akata-iCas9 cells. Knockdown of miR-141-3p was confirmed by qRT-PCR; miR-200c expression was not significantly affected (Fig. 5A and B; see also Fig. S2B and C). Cells were stimulated with anti-Ig for 48 h and protein levels subsequently analyzed by immunoblotting. Compared to control cells, basal levels of Foxo3a in miR-141 mutant cells were found to be markedly increased by 2- to 3-fold (Fig. 5C and D). BCR cross-linking substantially reduced Foxo3a in both control and miR-141 mutant cells (Fig. 5C and D), which coincided with upregulation of miR-141 (Fig. 5A and B); however, Foxo3a levels in the miR-141 mutant cells were increased compared to control cells. Thus, perturbation of miR-141 specifically enhances endogenous Foxo3a protein expression, supporting FOXO3 as a direct target of this miRNA in BL cells. Moreover, these experiments demonstrate that engagement of BCR signaling in lymphoma cell lines represses Foxo3a.

### Dynamics of Foxo3a expression following BCR stimulation.

**FIG 6:**
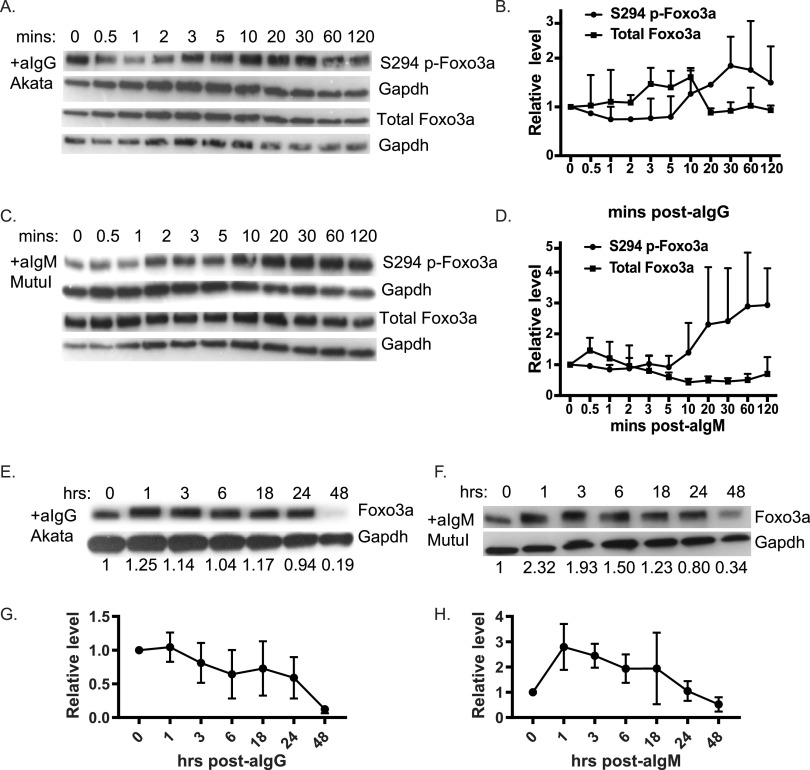
Foxo3a was phosphorylated and subjected to degradation in response to BCR cross-linking. (A and C) Time course of Ser294 phospho-Foxo3a and total Foxo3a protein levels in EBV-positive Akata cells treated with anti-IgG (A) or MutuI cells treated with anti-IgM (C). Gapdh levels are shown as loading controls. Shown are representative results of two to three independent experiments. (B and D) The band intensities in panels A and C were quantified using ImageJ, normalized to loading controls, and reported relative to levels at 0 min (*n* = 2). (E and F) Time course of Foxo3a protein levels in EBV-positive Akata cells treated with anti-IgG (E) or MutuI cells treated with anti-IgM (F). Significant reduction of total Foxo3a protein occurs at between 24 and 48 h. Shown are representative results of three independent experiments. (G and H) The band intensities in panels E and F were quantified using ImageJ, normalized to loading controls, and reported relative to levels at 0 min (*n* = 3).

To investigate the dynamics of Foxo3a repression during the EBV lytic infection cycle, we performed time course experiments and monitored protein levels at multiple times after anti-Ig treatment (Fig. 6). BCR engagement activates PI3K/Akt and Erk signaling pathways, which are required for EBV reactivation from latency (13). Moreover, these signaling pathways induce posttranslational modifications of Foxo3a which modulate its transcriptional activity, subcellular localization, and degradation (57). Erk inhibits Foxo3a activity via phosphorylation at Ser294, Ser344, and Ser425 which leads to Foxo3a degradation via MDM2 (58). We assessed Erk-mediated phosphorylation of Foxo3a (Ser294) in Akata and MutuI cells. Within 10 min of anti-Ig treatment, phosphorylated Foxo3a levels increased; however, total Foxo3a levels did not decrease, even by 2 h after BCR stimulation (Fig. 6A to D). We monitored total Foxo3a for longer times (Fig. 6E to H), and found that protein levels modestly increased early after anti-Ig treatment and then strongly declined between 24 and 48 h. This later time point corresponds to maximum induction of miR-141 and miR-BART9 (Fig. 2; see also Fig. S1B), supporting miRNA-mediated mechanisms as major contributors to Foxo3a suppression in EBV-infected BL cells.

### EBV miR-BART9 activity enhances Foxo3a repression and is necessary for a productive EBV lytic cycle.

**FIG 7:**
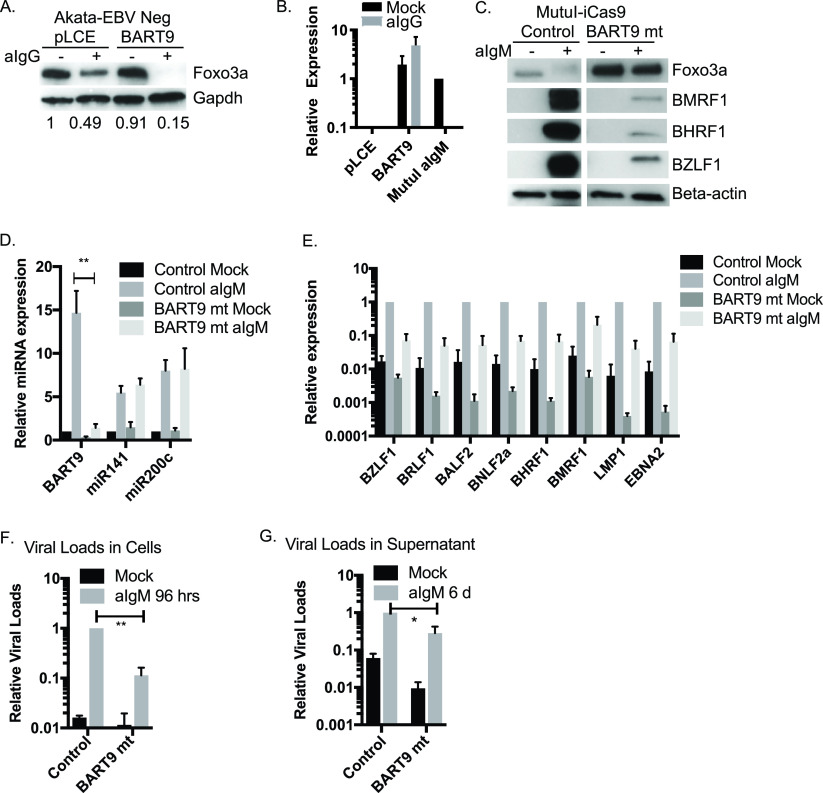
EBV miR-BART9 enhances Foxo3a inhibition. (A) miR-BART9 enhances Foxo3a suppression after surface Ig cross-linking. Akata-EBV Neg cells transduced with pLCE-miR-BART9 were treated with anti-IgG at 5 days postransduction. After 48 h of treatment, lysates were collected and subjected to immunoblotting as described above. Shown are representative results of three independent experiments. (B) TaqMan qRT-PCR analysis of miR-BART9 expression. EBV-negative Akata (Akata-EBV Neg) cells were transduced with pLCE-based miR-BART9 expression vectors. miR-BART9 expression values are normalized to cellular miR-16 and reported relative to levels in MutuI cells treated with anti-IgM for 48 h (*n* = 3). (C) Immunoblot of Foxo3a and lytic proteins in mock-treated or anti-IgM-treated MutuI-iCas9 single clones stably transduced with either empty gRNA (Control) or gRNA against miR-BART9 (BART9 mt). Beta-actin levels are shown as loading controls. Shown are the results of one of three representative and independent experiments. (D) TaqMan qRT-PCR analysis of miR-BART9 in MutuI-iCas9 confirms significant knockdown of miR-BART9, while homolog miRNAs miR-141 and miR-200c are not significantly impacted. Values are normalized to miR-16 and reported relative to levels of mock-treated empty gRNA control cells. **, *P* < 0.01 (Student *t* test). (E) qRT-PCR analysis of EBV viral gene expression. Expression levels are normalized to GAPDH and reported relative to anti-IgM-treated empty gRNA control cells. Student *t* tests were performed to compare expression of all eight genes in anti-IgM treated control and BART9 mt show significant differences (*P* < 0.01). (F and G) Genomic DNA (F) or supernatant (G) was collected from mock-treated or anti-IgM-treated MutuI-iCas9 single clones stably transduced with either empty gRNA or gRNA against miR-BART9. Viral loads were determined by qPCR assay using primers to the LMP1 region. For cell-associated viral loads (F), values are normalized to GAPDH. For viral loads in supernatant (G), values are determined by LMP1 standard curve. In both cases, values are reported relative to viral levels in anti-IgM-treated empty gRNA control cells. Shown are the averages from three independent experiments. *, *P* < 0.05; **, *P* < 0.01 (Student *t* test).

Given that miR-141 is consistently induced in response to BCR engagement irrespective of infection status, we wondered whether miR-BART9 might exert an additive effect in EBV-infected cells in suppressing Foxo3a following lytic reactivation. We therefore introduced miR-BART9 into EBV-negative Akata cells (Fig. 7A and B). Ectopic miR-BART9 did not impact basal Foxo3a; however, upon BCR cross-linking, we observed nearly total loss of detectable Foxo3a in the presence of miR-BART9 (Fig. 7A). These data indicate that the cellular environment conferred through BCR cross-linking, combined with the presence of EBV miR-BART9, plays a key part in Foxo3a regulation during EBV infection.

We next performed loss-of-function experiments using lentiviral gRNAs to disrupt endogenous miR-BART9 in MutuI-iCas9 cells. CRISPR-mediated inhibition of miR-BART9 (BART9 mt) resulted in significantly higher levels of total Foxo3a compared to control cells, consistent with miR-BART9 targeting of FOXO3 (Fig. 7C). To determine whether BART9 mt cells were responsive to reactivation stimuli, we treated cells with anti-IgM. BART9 mt cells exhibited high levels of Foxo3a and very little induction of IE and E proteins, indicating severe defects in the viral and cellular responses to BCR stimulation (Fig. 7C). Assessment of miR-141/200c induction revealed comparable cellular miRNA expression levels in BART9 mt compared to control cells, demonstrating that the cellular response to surface Ig cross-linking is still intact at some level (Fig. 7D). We measured IE, early, and late gene expression by qRT-PCR, and found that viral gene expression patterns in BART9 mt cells were attenuated across the board in both mock-treated and aIgM-treated cells (Fig. 7E). While it is possible that the attenuated EBV gene expression patterns could be due to loss of viral genomes from BART9 mt cells, cell-associated viral loads were not significantly different in BART9 mt cells compared to control cells (Fig. 7F, mock). Notably, we did observe reduced viral DNA copies and lower virus yields in BART9 mt cells following BCR stimulation (Fig. 7F and G). Together, these results demonstrate that miR-BART9 activity is necessary for Foxo3a suppression and productive lytic reactivation.

### FOXO3 inhibition promotes the EBV lytic cycle.

**FIG 8:**
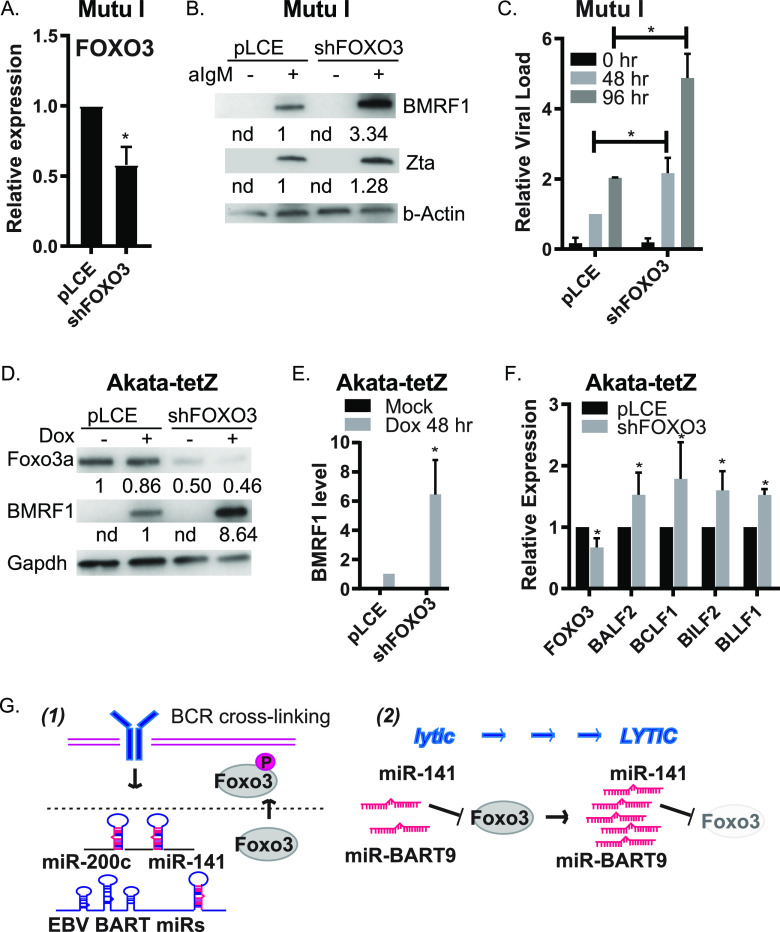
Posttranscriptional inhibition of FOXO3 augments the EBV lytic cycle. (A) MutuI cells were stably transduced with empty vector control (pLCE) or shRNA against FOXO3 and treated with anti-IgM for 48 h. Total RNA was collected and FOXO3 expression levels were determined by qRT-PCR. Values are normalized to GAPDH and shown relative to pLCE control cells. Shown are the averages from six independent experiments. *, *P* < 0.05 (Student *t* test). (B) MutuI cells stably transduced with vector control (pLCE) or shFOXO3 were treated with anti-IgM for 48 h, and lysates from mock-treated or anti-IgM-treated cells were collected. EBV BMRF1 and Zta proteins were detected by immunoblotting. Gapdh levels are shown as loading controls. Band intensities were quantified using ImageJ, normalized to loading controls, and reported relative to anti-IgM-treated empty vector cells. (C) Genomic DNA was isolated from MutuI cells stably transduced with pLCE or shFOXO3 and assayed by qPCR for viral loads. Values are normalized to GAPDH and reported relative to the levels of 48 h anti-IgM-treated empty vector cells. Shown are the averages from three independent experiments. *, *P* < 0.05 (Student *t* test). (D) Akata-tet-Z cells were stably transduced with empty vector control (pLCE) or shRNA against FOXO3 and treated with doxycycline for 48 h. Immunoblots were performed on lysates collected from mock- or doxycycline-treated cells for Foxo3a and BMRF1 lytic gene product. Shown are the results of one of three representative independent experiments. Gapdh levels are shown as loading controls. Band intensities were quantified using ImageJ, normalized to loading controls, and reported relative to mock-treated empty vector cells (Foxo3a) or doxycycline-treated empty vector cells (BMRF1). (E) Quantification of BMRF1 immunoblots. Reported are the averages from three independent experiments. *, *P* < 0.05 (Student *t* test). (F) BMRF1 target genes are upregulated upon FOXO3 inhibition. RNA was isolated from Akata-tet-Z cells at 48 h after Zta induction, and gene expression was assayed by qRT-PCR. Values are normalized to GAPDH and reported relative to levels of pLCE empty vector cells. Shown are the averages from five independent experiments. *, *P* < 0.05 (Student *t* test). (G) Proposed model of how BCR-responsive miR-141 and EBV miR-BART9 promote progression of the lytic cycle. (Panel 1) BCR-cross-linking induces phosphorylation of Foxo3a and expression of miR-141 and EBV miRNAs. (Panel 2) miR-141 and miR-BART9 suppress common host targets that include FOXO3. Posttranscriptional inhibition of Foxo3a enhances progression of EBV lytic reactivation.

Foxo3a levels inversely correlated with viral loads following reactivation (Fig. 7), suggesting that Foxo3a might be restrictive for progressive of the lytic cycle. To test this hypothesis, we implemented shRNAs to mimic miRNA activity and posttranscriptionally block FOXO3 expression in MutuI cells (Fig. 8A). FOXO3 knockdown resulted in significant increases in EBV BMRF1 levels upon anti-IgM stimulation (Fig. 8B). Significant increases in viral loads were further detected, demonstrating that posttranscriptional inhibition of FOXO3 sensitizes cells to reactivation stimuli (Fig. 8C). We carried out additional experiments in EBV-positive Akata-tet-Z cells in which the lytic cycle can be activated through Zta directly. Although miR-BART9 is expressed in these cells and accumulates in the presence of Zta, miR-141 is not induced in the absence of BCR stimulation (Fig. 1 and 2), thereby allowing us to uncouple direct EBV reactivation events from miR-141 and cell signaling effects initiated via the BCR. Consistent with results above, repression of FOXO3 significantly increased lytic BMRF1 antigen expression upon Zta induction (Fig. 8D and E). BMRF1 can function as a transactivator for a subset of EBV late genes (59). We detected moderate but significant increases in BALF2, BCLF1, BILF2, and BLLF1 by qRT-PCR in shFOXO3 cells (Fig. 8F), congruent with enhanced BMRF1 levels. While further mechanistic studies will need to be performed to determine exactly how FOXO3 controls the lytic reactivation process, these data demonstrate that RNAi-mediated translational repression of FOXO3 can phenocopy the functional effects of BCR-mediated miR-141 induction and augment the EBV lytic replication cycle.

Taken together, our experiments support a model whereby BCR engagement triggers transcriptional activation of miR-141/200c, as well as Erk-mediated phosphorylation of Foxo3a (Fig. 8G). In EBV-infected cells, BCR engagement further activates lytic replication, leading to accumulation of viral miRNAs such as miR-BART9. Through seed-sequence mimicry, multiple targets of miR-141-3p are cotargeted by miR-BART9-3p, and although several identified miRNA targets (ZEBs, YY1, and RANBP9) have previously described roles in repressing reactivation in epithelial cells, we demonstrate here that one common target of these miRNAs, FOXO3, restricts the lytic phase in BL cells. Thus, while miR-141 induction and FOXO3 suppression are physiological cellular responses to BCR triggers, EBV further harnesses and boosts this response through virally encoded miR-BART9 to efficiently promote the lytic cycle.

## DISCUSSION

In this study, we addressed the involvement of cellular miRNAs in EBV reactivation in B cells. Through in-depth evaluation of miRNA expression, we found that miR-141, a member of the miR-200 family, was significantly induced in both EBV-positive and -negative BL cells in response to BCR stimulation. Interestingly, genetic disruption of miR-141 detrimentally impacted virus reactivation, suggesting that miR-141 and the direct targets of this miRNA are key factors in the EBV lytic replication cycle.

The functions of miR-200 family members in B cell processes, and specifically, EBV replication in B cells, are not fully known, which may be due in part to their complex expression patterns in B cell subsets. Initial miRNA sequencing studies detected miR-200 family members in naive and memory B cells but not in GC or plasma cells (60). miR-200 family members were found to be increased in tonsillar B cells compared to normal, resting CD19^+^ B cells, and B lymphoma cells (AIDS-DLBCL, as well as non-AIDS DLBCL) (61). Moreover, analysis of biopsy samples from a cohort of 83 DLBCL patients suggested loss of miR-200c was linked to poor prognosis (61). Recent studies showed that miR-200a, miR-200b, miR-200c, miR-429, and miR-141 are upregulated in CD77^–^ B cells (centrocytes and plasmablasts) compared to naive cells and centroblasts (62). Thus, the presence of miR-200 family members is tightly linked to specific stages of B cell development and differentiation. Our experiments imply that surface Ig expression and triggering of BCR response pathways could mechanistically explain the different levels of miR-141 and miR-200c detected in these prior studies.

Accumulating evidence indicates that miR-141 and other miR-200 family members play crucial roles in the replication of both DNA and RNA viruses. Picornaviruses, such as enterovirus 71, induce miR-141 and miR-200c through EGR1; miR-141-3p subsequently targets eIF4E to aid in host shutoff, thereby positively impacting productive virus replication (43). Through the use of shRNAs, we show here that EGR1 is also partially responsible for miR-141 induction in BL cells. In other virus systems, miR-141 may be detrimental to virus replication. For example, introduction of artificial miR-141 mimics into HepG2 cells interferes with hepatitis B virus replication (63). For herpesviruses, human cytomegalovirus (HCMV) harbors binding sites for miR-200b/c and miR-429 in the 3′ UTR of the IE gene UL122 (encoding IE2) and mutational inactivation of the miRNA binding site leads to increased viral loads (64). Thus, miR-200 family members negatively impact HCMV replication, potentially during acute infection or reactivation from latency, by interfering with expression of the lytic cascade. In contrast, for EBV-infected epithelial cells, miR-200b/c and miR-429 have been shown to suppress transcriptional repressors (ZEBs) of the EBV IE promoters, thereby indirectly enhancing IE gene expression (22, 23). We found that miR-141 could target the ZEB2 3′ UTR and reduce Zeb2 levels in HEK293T cells. However, consistent with several studies examining Zeb expression in lymphocytes and other immune cell populations (65), we were unable to confirm Zeb2 expression in BL cells, thus prompting us to explore roles for other miR-141 targets in EBV reactivation.

Target identification for miR-141 in EBV-infected cells is complicated by the fact that EBV encodes a viral miRNA (miR-BART9-3p) with seed sequence homology to miR-141-3p. Although it has long been hypothesized that this viral miRNA might act as a functional mimic of miR-141 and/or other miR-200 family members, we formally demonstrate in this study that this is indeed the case. Using luciferase reporter assays, we show that multiple cellular 3′ UTRs can be commonly targeted by both miR-141 and miR-BART9. In addition, RanBP9 and Foxo3a protein levels were suppressed in the presence of either miRNA. Intriguingly, we identified two 3′ UTRs (YY1 and CCDC6) that responded only to miR-141, despite strong seed matches to miR-BART9-3p. Furthermore, Zeb2 levels were inhibited by miR-141 but not miR-BART9, despite the 3′ UTR reporter responding to both miRNAs. While it remains to be specifically tested, these data strongly suggest that base pairing outside the seed region dictates the exact repertoire of targets for each miRNA and also contributes to the overall level of translational repression for each of their targets.

Given the potent inhibition of Foxo3a levels upon miR-141 or miR-BART9 expression, we selected this target as a potential candidate for involvement in EBV lytic replication. Foxo transcription factors have a wide range of functions and participate in a multitude of cellular processes, including cell cycle, apoptosis, stress response, and cell differentiation (51, 57, 66, 67). Notably, chemical inhibition of one member of the Foxo family, Foxo1, induces reactivation of the related human gammaherpesvirus, Kaposi’s sarcoma herpesvirus, in adherent iSLK cells (68), suggesting an important role for Foxo proteins in modulating the herpesvirus latent-to-lytic switch. Very recent work from Hancock et al. demonstrated that two HCMV miRNAs, miR-US5-1 and miR-UL112-3p, downregulate Foxo3a in CD34^+^ hematopoietic cells, thereby protecting cells from apoptosis (69).

An understanding of the role of FOXO3 in B cell functions and EBV biology remains incomplete. Studies in murine models have shown that PI3K/Akt/Foxo signaling contributes to multiple aspects of lymphocyte development, including control of GC transcriptional programs and regulation of AID in activated B cells (66, 70). The majority of studies to date have focused on FOXO1, which is induced by EBF1, TCF3, and other B cell transcription factors, and plays an essential role in pro- and pre-B cell development (reviewed in reference 51). FOXO3 transcripts are potently induced in human B cells committed to plasma cell differentiation (67). Posttranslational modifications such as phosphorylation, acetylation, and glycosylation control subcellular localization and transcriptional activity of Foxo proteins (57, 71). Cytoplasmic, phosphorylated Foxo3a is marked for degradation by the ubiquitylation-mediated proteasome degradation pathway, while interactions between Foxo3a and 14-3-3 proteins result in nuclear shuttling (57, 71). In the context of EBV infection, LMP1 can induce Akt activation and phosphorylation of Foxo3a, resulting in Foxo3a translocation out of the nucleus (72). Here, we demonstrate that the overall levels of Foxo3a are additionally controlled through both viral and host miRNA-mediated posttranscriptional regulation of FOXO3 transcripts. Through loss-of-function experiments, we further establish that FOXO3 inhibition positively impacts EBV replication and enhances expression of BMRF1, an early lytic protein that functions as both a transcriptional activator and a viral DNA polymerase processivity factor. Exactly how FOXO3 regulates BMRF1 is currently unknown. FOXO3 does not appear to significantly impact Zta protein expression, but does alter expression of EBV late genes that are transactivated by BMRF1, suggesting that RNAi-mediated suppression of FOXO3 functionally impacts the later stages of EBV lytic replication. This action would correlate with the timing of FOXO3 downregulation which we find to occur at least 24 h after BCR cross-linking. Further mechanistic studies will be needed to unveil exactly how FOXO3 regulates the EBV lytic cascade, as well as associated cellular factors, in order to understand how these factors coordinate to functionally impact the critical transitions from latency to reactivation and productive lytic replication.

Interestingly, in other virus infection systems, FOXO3 suppression is linked to antiviral responses. FOXO3 blocks transcription of IRF7 in macrophages in response to poly(I⋅C), and FOXO3 knockout mice exhibit increase lung injury during vesicular stomatitis virus infection (73). Type I interferon (IFN) can suppress FOXO3 levels (73), and more recent studies show this occurs partly through activation of miR-223 which targets the FOXO3 3′ UTR (74). Of note, we identified another common target of miR-141 and miR-BART9, ZCCHC3, that functions as a cosensor for cGAS, activates the IFN-β promoter, and contributes to innate immune responses directed against DNA virus infection in the cytosol (54). Overexpression of ZCCHC3 enhances herpes simplex virus 1-mediated activation of IFN response genes (54). Thus, regulation of FOXO3 and ZCCHC3 levels by miR-141 and miR-BART9 may play an important role in modulating innate immune responses activated during EBV replication.

In summary, these data enhance our current understanding of how host and viral miRNAs contribute to the EBV lytic cycle. To date, the majority of studies have pointed to roles for miRNAs in supporting latent infection for herpesviruses (75–77). Against convention, we demonstrate that specific miRNAs can also act to promote progression of the lytic cycle, lending support to the idea that these molecules actively orchestrate aspects of latency, reactivation, and lytic replication to cooperatively facilitate viral persistence within a host. Future work is needed to understand the specific relationships between miR-141 and miR-BART9 coregulated factors which can reveal novel therapeutic targets for EBV-associated lymphomas.

## MATERIALS AND METHODS

### Cell culture.

BL cell lines were maintained at 37°C in a 5% CO2-humidified atmosphere in RPMI 1640 supplemented with 10% fetal bovine serum (FBS) and 1% penicillin, streptomycin, and l-glutamine (P/S/G). MutuI cells originated from the laboratory of Erik Flemington. EBV-negative Akata cells were provided by Renfeng Li. Akata-tet-Z cells were maintained in tetracycline-free media and provided by J. J. Miranda with permission from Alison Sinclair. HEK293T and 293-2089 cells were maintained in high-glucose Dulbecco modified Eagle medium supplemented with 10% FBS and 1% P/S/G. For preparation of lentiviruses, HEK293T cells were plated in 15-cm plates in complete media and transfected using polyethylenimine (PEI) with 15 μg of lentivector, 9 μg of pDeltaR8.75, and 6 μg of pMD2G. Medium was changed to complete RPMI 1640 at between 8 and 16 h posttransfection. Lentiviral particles were harvested by sterile filtration of the supernatant using a 0.45-μm filter at 48 and 96 h posttransfection and used to transduce ca. 1 × 10^6^ to 5 × 10^6^ cells. For BCR cross-linking, BL cells were spun down and plated at 0.5 × 10^6^ cells in fresh medium containing soluble anti-IgM or anti-IgG (Sigma) at the concentrations and times indicated in the figure legends (2.5 to 5 μg/ml for 22 to 48 h).

### Plasmids.

pLCE-based miRNA expression vectors contain ∼200 nt of the pre-miRNA as previously described (45). Functional miRNA expression was confirmed by indicator assays as previously described (45). The miR-141 indicator is from Addgene (catalog no. 67632), and the shRNAs against FOXO3 and EGR1 were gifts from Jay Nelson’s laboratory at the Vaccine and Gene Therapy Institute. To generate 3′ UTR luciferase reporters for BCL6, CDK6, IKZF2, MCL1, ZCCHC3, and ZEB2, regions were PCR amplified from genomic DNA of EBV-infected B cells and cloned into the XhoI and NotI sites downstream of *Renilla* luciferase in the psiCheck2 dual luciferase reporter vector containing an expanded multiple cloning site (78). psiCheck2 3′ UTR constructs for FOXO3, RANBP9, and YY1 were provided by Jay Nelson’s laboratory. Additional 3′ UTR reporters are cloned into pLSG as previously described (31, 45). Oligonucleotide sequences used for cloning are available upon request. Mutant 3′ UTR reporters, containing nucleotide changes in miRNA seed match sites as identified by PAR-CLIP, were generated by Phusion *Taq* site-directed mutagenesis as previously described (79).

### CRISPR editing.

Inducible Cas9 (iCas9) BL cells were established by transducing cells with pCW-Cas9-BLAST-based lentiviruses (Addgene, catalog no. 83481) and selecting with Blasticidin. iCas9 cells were subsequently transduced with LentiGuide-puro lentiviral vector (Addgene, catalog no. 52963) bearing either empty guide RNA (gRNA) as control or gRNA against miR-141 or miR-BART9 and then selected with puromycin. Stable cell lines were treated with doxycycline for 7 days prior to analysis. Cells were treated with anti-IgM for 48 h and lysates harvested for immunoblot analysis. miR-BART9 mutant MutuI single clones were isolated by plating stable cell line at 0.5 cell/well onto 96-well plates and culture for at least 2 weeks until single clones expanded. Isolated single clones were subjected to anti-IgM treatment for 48 h, and total RNAs were extracted. TaqMan miRNA qRT-PCR analysis was conducted to select for the single clone with the most significant miR-BART9 knockdown efficiency.

### Quantitative RT-PCR and PCR analysis.

For gene expression analysis, total RNA was extracted using TRIzol (Thermo Fisher), DNase-treated, and reversed transcribed using MultiScribe (Thermo Fisher) with random hexamers. Cellular and viral genes were detected using PowerUp SYBR green qPCR (Thermo Fisher). Oligonucleotides sequences are available upon request. For cell-associated viral loads, genomic DNA was isolated using DNAzol (Thermo Fisher). Then, 100 ng of DNA was analyzed using primers to the LMP1 region and normalized to GAPDH (glyceraldehyde-3-phosphate dehydrogenase) levels, as previously described (31). For viral loads in supernatant, cells were treated with anti-Ig for 18 h. Cells were washed by PBS and replated in RPMI 1640 complete medium at a 2 × 10^6^/ml density. Four days later, supernatant was collected and filtered with 0.45-μm polyvinylidene difluoride filters (Millipore) and concentrated by Amicon Ultra-4 centrifugal filter units. Virion DNA was extracted from concentrated supernatant according to a standard protocol from Promega. Viral loads were determined by qPCR for LMP1 copies using LMP1 standard curve. All PCRs were performed in technical replicates (duplicates or triplicates).

### 3′ UTR reporter assays.

HEK293T cells plated in 96-well black-well plates were cotransfected with 20 ng of 3′ UTR reporter and 250 ng of control vector (pLCE) or miRNA expression vector using Lipofectamine 2000 (Thermo Fisher). At 48 to 72 h posttransfection, the cells were harvested in 1× passive lysis buffer (Promega), and lysates were assayed for dual luciferase activity using a dual luciferase reporter assay system (Promega) and a luminometer. All values are reported as relative light units (RLU) relative to luciferase internal control and normalized to pLCE control vector.

### Immunoblotting.

Cells were lysed in NP-40 lysis buffer (50 mM HEPES [pH 7.5], 150 mM KCl, 2 mM EDTA, 1 mM NaF, 0.5% [vol/vol] NP-40, 0.5 mM dithiothreitol). Protein concentrations were determined using the bicinchoninic acid protein assay kit (Thermo Scientific), and 20 μg of total protein lysate was resolved on 10% Tris-glycine SDS-PAGE and then transferred onto Immobilon polyvinylidene difluoride membranes. Blots were probed with primary antibodies to Zeb2 (sc-271984; Santa Cruz), Foxo3a (2497S; Cell Signaling), phospho-Foxo3a Ser294 (5538S; Cell Signaling), RanBP9 (17755-1-AP; Proteintech), EBV Ea-D (BMRF1, sc-0261; Santa Cruz), EBV Zebra (BZLF1, sc-53904; Santa Cruz), BHRF1 (biorbyt orb518144), GAPDH (sc-47724; Santa Cruz), or beta-actin (sc-47778; Santa Cruz), followed by horseradish peroxidase-conjugated secondary antibodies (anti-rabbit IgG or anti-mouse IgG). Blots were developed with enhanced chemiluminescent substrate (Pierce). Band intensities were quantified using ImageJ, normalized to loading controls, and reported relative to control cells.

### miRNA deep sequencing and bioinformatics.

miR-Seq libraries were generated from total RNA using the Illumina small RNA TruSeq kit as per manufacturer’s recommendations and sequenced multiplexed on the Illumina MiSeq at the ONPRC Molecular Biology Core. Prior to library preparation, purity of input RNA was assessed using a NanoDrop 2000 spectrophotometer (Thermo Scientific) and OD260/280 ratios of 1.8 to 2.1 were considered acceptable. Raw sequencing reads obtained in FASTQ format were preprocessed to remove linkers and aligned concurrently to the human genome (hg19) and MutuI EBV genome (KC207814.1) using Bowtie (v1.0.1 http://bowtie-bio.sourceforge.net/index.shtml) (-v 2 –m 10) (80). miRNAs were annotated and quantified by miRDeep (81). EdgeR (82) was used to define the significant, differentially expressed miRNAs in control versus anti-IgM treated cells (*P* < 0.05, FDR < 0.05, read counts >20). Heatmaps were generated in R. Raw miR-Seq data files can be accessed through NCBI short read archive (SRA; BioProject ID PRJNA596463, BioSample accession numbers SAMN13624391, SAMN13624392, SAMN13624393, and SAMN13624394).

### Statistical analyses.

Luciferase and PCR data are reported as the means from at least three independent experiments (unless otherwise stated) with the standard deviations (SD). Statistical significance was determined by using a paired Student *t* test, performed with Microsoft Excel 2010, and *P* values of <0.05 were considered significant.
